# TIDieR-PHP: a reporting guideline for population health and policy interventions

**DOI:** 10.1136/bmj.k1079

**Published:** 2018-05-16

**Authors:** Mhairi Campbell, Srinivasa Vittal Katikireddi, Tammy Hoffmann, Rebecca Armstrong, Elizabeth Waters, Peter Craig

**Affiliations:** 1 MRC/CSO Social and Public Health Sciences Unit, University of Glasgow, 200 Renfield Street, Glasgow G2 3QB, UK; 2Centre for Research in Evidence-Based Practice, Bond University, Queensland, Australia 3Melbourne School of Population and Global Health, University of Melbourne, VIC, Australia; * Deceased 22 September 2015

## Abstract

We lack guidance on how to describe population health and policy (PHP) interventions in reports of evaluation studies. PHP interventions are legal, fiscal, structural, organisational, environmental, and policy interventions such as the regulation of unhealthy commodities, health service reorganisation, changes in welfare policy, and neighbourhood improvement schemes. Many PHP interventions have characteristics that are important for their implementation and success but are not adequately captured in the original Template for Intervention Description and Replication (TIDieR) checklist. This article describes the development of a revised reporting template for PHP interventions (TIDieR-PHP) and presents the checklist with examples for each item

**Summary points**

Inadequate descriptions of interventions are a source of waste in health research
Existing reporting guidelines do not adequately describe key characteristics of population health and policy interventions
TIDieR-PHP provides a template for reporting public health, health systems, and social and environmental policy interventions

Population health and policy (PHP) interventions are delivered across communities and may have large effects on population health and health inequalities.^1^ ^2^ ^3^ They include legal, fiscal, structural, organisational, environmental, and policy interventions that seek to change health related behaviours or to modify the social and economic determinants of health and interventions with other goals that bring about such changes as a by product. Examples include legislation for smoke-free public places; reduced urban speed limits; gun control laws; regulation of advertising; welfare reform; conditional cash transfers; health system reorganisation; neighbourhood regeneration; taxation of tobacco, alcohol, and sugar sweetened beverages; international trade agreements; and health information and promotion campaigns. A key feature of these interventions is that they are delivered collectively to whole populations rather than to individuals.^4^

Inadequate description of interventions in evaluation reports is a major source of waste in research.^5^ Unclear or limited information on interventions impedes evidence synthesis, leads to unnecessary research duplication, and hinders implementation in other populations or settings. Reporting guidelines for clinical trials (CONSORT^6^) and other evaluation designs (such as STROBE^7^ and TREND^8^) include items related to intervention description but provide little explicit guidance. The Template for Intervention Description and Replication (TIDieR) checklist^9^—which is an extension of the CONSORT 2010^6^ (item 5) and SPIRIT 2013^10^ (item 12) checklists—has 12 items and is designed to improve the completeness of reporting and the replicability of interventions. It has been used by many trial authors, mandated by numerous journals, and is required by the Cochrane Collaboration for systematic reviews and some trial registries.

The original version of TIDieR is not able to provide checklist items suitable for all types of interventions but has scope for adaptation.^9^ Many PHP interventions have characteristics that are not captured clearly by the existing TIDieR items. For example, information such as any underpinning legislation, regulation of the intervention, or organisations involved in implementing policy for a PHP intervention may be important for understanding how the intervention works and whether it would be appropriate in other circumstances. PHP interventions can be developed differently from experimental interventions; some are natural experiments, in which control of the intervention is not held by the investigator, and many are unlikely to be directly replicated. A precise description of the intervention is required to enable other investigators or policy makers to interpret the intervention to fit their situation. The details of the design and implementation of PHP interventions have a substantial affect on health outcomes. Comprehensive smoke-free legislation, for example, has a much stronger effect than partial smoke-free legislation.^11^ This paper describes the development and final version of TIDieR-PHP—an adaption of the original TIDieR checklist for reporting PHP interventions.

## Methods

The TIDieR-PHP project team comprised six members, all with experience of conducting, evaluating, or reviewing PHP interventions; TH was lead author of the original TIDieR guideline. We found no guidelines for reporting PHP interventions published or in development in the Equator Network library. We undertook an assessment exercise with a sample of PHP intervention studies to establish what information is not captured, or not captured well, by the original TIDieR checklist. Using an iterative process of incorporating these characteristics and testing them against additional interventions, we created a draft reporting checklist. We conducted a two round Delphi survey of participants selected for their expertise in developing or assessing PHP interventions, including journal editors, funders, and PHP researchers. After this, the project team met to discuss the second round Delphi results and finalise the TIDieR-PHP checklist and item wording. We then developed and refined the accompanying guide. Full details of the methods used are provided in supplementary file 1.

## Results

### TIDieR-PHP checklist explanation and elaboration

A complete list of checklist items is provided in table 1, and an online version is available at www.equator-network.org/reporting-guidelines. An explanation and elaboration for each TIDieR-PHP checklist item is provided below. Examples and citation details are provided in supplementary file 2.

**Table 1:** TIDieR-PHP items with examples. TIDieR-PHP is intended to complement and be used as an extension to the appropriate reporting guideline for the study design being used (such as CONSORT, SPIRIT, STROBE, or TREND)

Item	Item description	Page in manuscript where item is reported	Other*
1 Brief name	Provide the name or a phrase that describes the intervention		
2 Why	Describe the logic, mechanisms, or rationale of the intervention, clearly linking intervention elements to the expected effects on immediate or longer term outcomes (or both)		
3 What materials	Describe any materials used in the intervention (including online appendices or URLs for further details). For example: —informational materials (may include those provided to recipients of the intervention or in training of intervention providers) —nature and value of any benefit provided (eg, cash, voucher, meal) —any physical resources or infrastructure provided as part of the intervention		
4 What and how	Describe how the intervention was planned, established, and intended to be delivered. Depending on the type of intervention, it may be useful to consider: —how sources of funding for the intervention and the service providers were obtained, how users were enrolled and the service delivered —how any payments were made or benefits delivered, how qualifying conditions were implemented —the entity being regulated, the scope of the regulation, permitted level of use; procedures for monitoring or enforcing compliance, and any sanctions for non-compliance —how people were exposed to the intervention, whether it was provided to individuals or larger populations —any underpinning legislation including name, date passed, and legislative body		
5 Who provided	Describe the provider of the intervention, including legal status and powers, field organisations and staff responsible for planning, implementation, monitoring and enforcement. Where relevant, describe intervention provider expertise and training (general or specific to the intervention)		
6 Where	Describe the type of location (eg, school, community centre) and the geographical scope of the intervention (eg, national, regional, city-wide). Where relevant, describe the historical, cultural, socioeconomic, or political background to the intervention		
7 When and how often	Describe when the intervention was implemented, how long it remained in place, and, if applicable, the number, duration, and scheduling of occasions		
8.1 Planned variation	Describe and provide the reason for any variation or tailoring that was planned or allowed for in the design of the intervention. Examples include differences between locations, geographical areas, population subgroups, or over time		
8.2 Unplanned variation	Describe and provide the reason for any unplanned variation or modifications in the intervention (eg, between different locations, geographical areas, population subgroups, or over time) that were made after the intervention commenced		
9.1 How well	Describe any strategies used or actions taken to maintain fidelity of the intervention (ie, to ensure that the intervention was delivered as intended)		
9.2 How well—delivery	Describe the fidelity of the intervention (ie, the extent to which the intervention was delivered as intended)		

* If the information is not provided in the primary paper, give details of where this information is available (eg, protocol, other published papers (provide citation details), or a website (provide the URL))

#### Item 1: Brief name

*Description*—Provide the name or a phrase that describes the intervention.

*Explanation*—Clarity in the name of the intervention, or a brief description contained within the name, allows users to identify the type of intervention and enables multiple reports of the same intervention to be recognised. The intervention name should be clear and simple (example 1a), and acronyms should be provided in full (example 1b).

#### Item 2: Why

*Description*—Describe the logic, mechanisms, or rationale of the intervention, clearly linking intervention elements to the expected effects on immediate or longer term outcomes (or both).

*Explanation*—Providing the underlying rationale of the intervention enables readers to understand its essential components. This is particularly important when the intervention is complex. The underlying rationale or logic should explain how the components or mechanisms link to the intended outcomes. The rationale may focus on identifying the mechanisms expected to produce the intended outcomes (example 2a) or focus on the underlying logic of how the intervention is expected to result in the intended outcomes (example 2b).

#### Item 3: What materials

*Description*—Describe any materials used in the intervention (including online appendices or URLs for further details). For example, informational materials (may include those provided to recipients of the intervention or in training of intervention providers); nature and value of any benefit provided (eg, cash, voucher, meal); any physical resources or infrastructure provided as part of the intervention.

*Explanation*—Any physical or informational materials used for the intervention should be described (example 3a). Full descriptions or supplementary information could be provided as an online appendix or in the study protocol. In some PHP interventions, “materials” may be more than physical and informational. The checklist item provides examples to encourage authors to consider broadly the range of physical materials used in the intervention. Examples 3b and 3c describe the type, amount, and increment schedule of the incentive payments central to their interventions. Example 3d provides details of how structural changes are implemented as part of the materials component of the intervention.

#### Item 4: What and how

*Description*—Describe how the intervention was planned, established, and intended to be delivered. Depending on the type of intervention, it may be useful to consider: how sources of funding for the intervention and the service providers were obtained, how users were enrolled and the service delivered; how any payments were made or benefits delivered, how qualifying conditions were implemented; the entity being regulated, the scope of the regulation, permitted level of use; procedures for monitoring or enforcing compliance, and any sanctions for non-compliance; how people were exposed to the intervention, whether it was provided to individuals or larger populations; any underpinning legislation including name, date passed, and legislative body.

*Explanation*—The concepts of what and how PHP interventions are provided are strongly interwoven. The procedures or activities of the intervention should be described, including processes crucial to setting up the intervention, sources of funding (example 4a), all stages of operating the intervention, and how people were exposed to it (example 4b). Other features that may be relevant include how any payments or sanctions were applied (example 4c). The policy background of PHP interventions is often important, as the effects of the intervention may depend on regulation or legislation. Where relevant, therefore, descriptions should be provided of the entity being regulated (example 4d) or any legislation underpinning the intervention (example 4e).

#### Item 5: Who provided

*Description*—Describe the provider of the intervention, including legal status and powers, field organisations and staff responsible for planning, implementation, monitoring and enforcement. Where relevant, describe intervention provider expertise and training (general or specific to the intervention).

*Explanation*—Although PHP interventions may be delivered by a single organisation, often a broad range of individuals and organisations are involved (examples 5a and 5b). A clear description of the field organisations, regulators, and legislative bodies involved in the delivery and oversight of the intervention is important for understanding the reach and sustainability of the intervention. The description should include details of any legal (or similar) powers under which these bodies are operating (example 5c). The expertise of staff who deliver the intervention should be noted where this has implications for the quality of implementation and cost of delivering the intervention (example 5d).

#### Item 6: Where

*Description*—Describe the type of location (eg, school, community centre) and the geographical scope of the intervention (eg, national, regional, city-wide). Where relevant, describe the historical, cultural, socioeconomic, or political background to the intervention.

*Explanation*—The effects of an intervention depend not just on its component parts but on how those components interact with its organisational and geographical setting and the wider historical, cultural, socioeconomic and political context. Geographical setting should always be specified (example 6a). There is a wide range of dimensions of context (example 6b), so attention should be focused on those most likely to explain variation in implementation, uptake, or outcomes.^12^

#### Item 7: When and how often

*Description*—Describe when the intervention was implemented, how long it remained in place, and, if applicable, the number, duration, and scheduling of occasions. 

*Explanation*—Reporting when the intervention was implemented and how long it was operative for is useful for understanding the amount and intensity of exposure to the intervention (example 7a) and for indicating when effects might start or cease (example 7b). For policy interventions, a clear indication of the implementation date and duration also enables readers to consider the possibility of confounding by concurrent policy initiatives.

#### Item 8.1: Planned variation

*Description*—Describe and provide the reason for any variation or tailoring that was planned or allowed for in the design of the intervention. Examples include differences between locations, geographical areas, population subgroups, or over time.

*Explanation*—This item captures variation in the intervention that arises from decisions made before implementation begins. It includes variations that are “allowed” because of differences that may occur in participant populations or setting characteristics. Some PHP interventions are designed to give local decision makers flexibility to tailor the intervention to local needs or circumstances (examples 8.1a-c). Distinguishing between variation arising from such flexibility and departures from the intervention protocol (item 8.2) is important.

#### Item 8.2: Unplanned variation

*Description*—Describe and provide the reason for any unplanned variation or modifications in the intervention (eg, between different locations, geographical areas, population subgroups, or over time) that were made after the intervention commenced.

*Explanation*—Unplanned variation refers to unforeseen changes to the intervention or departures from the intervention protocol that occur after implementation has begun. Unplanned variation may imply a loss of fidelity (example 8.2a) or may reflect modification in response to changes in policy (8.2b) or emerging information (8.2c). Unplanned variation in delivery or modification of the intervention is important to understanding variation in outcomes between sites or over time, difficulties with implementation, and, in some cases, possible solutions.

#### Item 9.1: How well

*Description*—Describe any strategies used or actions taken to maintain fidelity of the intervention (ie, to ensure that the intervention was delivered as intended).

*Explanation*—Interventions may depart from what was planned or intended in ways that have the potential to substantially influence the effects observed in an evaluation study. Understanding actions undertaken by those responsible for the intervention (or by other relevant people) to maintain intervention fidelity is important for future replication studies, to ensure that the intervention is delivered in a comparable way, and for ensuring that the integrity of the intervention is maintained under larger scale implementation. Measures to ensure fidelity include performance monitoring or setting targets (example 9.1a) or actions taken to increase the quality of the delivery (example 9.1b).

#### Item 9.2: How well—delivery

*Description*—Describe the fidelity of the intervention (ie, the extent to which the intervention was delivered as intended).

*Explanation*—This item captures details about whether the intervention was delivered as planned. This is important for distinguishing between intervention failure and implementation failure, explaining variation in effectiveness across sites, and identifying whether and how delivery needs to be improved. This information may be gathered via a process evaluation or from monitoring data, which may be qualitative or quantitative (example 9.2a, 9.2b, 9.2c).

## Discussion

We developed the TIDieR-PHP checklist to enable clear and comprehensive reporting of PHP interventions. It provides a brief list of items to capture pertinent features of these interventions. Precise descriptions are crucial to understanding implementation. TIDieR-PHP was produced in consultation with researchers, methodologists, journal editors, and funders experienced in conducting studies of and reviewing PHP interventions. It retains a similar structure to the original TIDieR guidelines but extends the scope to PHP interventions that are not fully covered by TIDieR.

We encourage users to consider the intervention to be reported in relation to the original TIDieR guidelines and this adaptation to decide which guideline is best suited. TIDieR-PHP is not intended to replace the original TIDieR checklist but is a reporting tool to guide the user to consider features of policies, legislations, and other population level interventions. We envision that TIDieR-PHP will be used in conjunction with other checklists, such as those for study reporting that don’t directly refer to describing interventions (eg, STROBE^13^ or RECORD 14) and those that mention intervention reporting in just one item (eg, CONSORT,^6^ SPIRIT,^10^ and TREND^8^). Guidelines with a stronger emphasis on describing interventions have been created for specific categories, such as the guide for reporting nursing interventions developed by Conn and Groves,^15^ the WIDER (Workgroup for Intervention Development and Evaluation Research) recommendations for reporting behaviour change interventions, which focuses on clinical interventions,^16^ and CReDECI 2 (Criteria for Reporting the Development and Evaluation of Complex Interventions in healthcare).^17^ TIDieR-PHP fills the gaps in these guidelines for PHP interventions.

## Conclusion

Inadequate description of interventions is a large source of waste in health research. Existing reporting guidelines are not designed to fully capture key characteristics of PHP interventions. We developed TIDieR-PHP to supplement existing reporting guidelines and to provide guidance to improve the reporting of PHP interventions. The guideline will help evaluation researchers describe PHP interventions clearly, by drawing attention to the characteristics that should be reported. Clear description of PHP interventions will help other researchers, policy makers, and practitioners to interpret evaluation studies, develop replication studies, and use the evidence for decision making. Editors should consider incorporating TIDieR-PHP in journal guidelines for authors and reviewers. Using TIDieR-PHP for clear reporting of PHP interventions will increase the sharing of knowledge and maximise the use of population health research.

## Web extra

Extra material supplied by authors

**Supplementary file 1**: Methodology for developing TIDieR-PHP

**Supplementary file 2**: Population health and policy intervention examples and citation details

## Notes

Ethical approval: Ethical approval for the Delphi survey was obtained from the University of Glasgow College of Social Sciences Ethics Committee.

Transparency: MC affirms that the manuscript is an honest, accurate, and transparent account of the study being reported; no important aspects of the study have been omitted; and any discrepancies from the study as planned (and, if relevant, registered) have been explained.
