# Supplementary material for: TIDieR-PHP: a reporting guideline for population health and policy interventions
Source: BMJ. 2018 May 16;361:k1079. doi: 10.1136/bmj.k1079 (PMC5954974; doi:10.1136/bmj.k1079)
Supplement: Supplementary file 1 — Methodology for developing TIDieR-PHP [file camm041840.ww1.pdf]

TIDieR-PHP: a reporting guideline for population health and policy interventions, explanation and elaboration

Supplementary file 1: Methodology for developing TIDieR-PHP

Below we report the methods used to develop the TIDieR-PHP checklist. The TIDieR-PHP project team comprised six members, all with experience of conducting, evaluating or reviewing PHP interventions; TH was lead author of the original TIDieR guideline. We were guided by the recommendations for developing health research guidelines [1].

Currently available guidance for describing interventions

We began by reviewing the Equator Network library of reporting guidelines to ensure there were no guidelines published or in development for reporting PHP interventions. The CONSORT guideline for reporting randomised controlled trials has one item referring to the description of the intervention [2]. The guideline for reporting study protocols, SPIRIT, states "*Interventions for each group with sufficient detail to allow replication, including how and when they will be administered*." [3]. Likewise, the TREND statement, (Transparent Reporting of Evaluations with Nonrandomized Designs) for behavioural and public health interventions which provides guidance for reporting an entire study [4], provides eight guiding statements to help describe the intervention. However, only one of these asks for description of what was provided as the intervention. The descriptors tend to focus on individual/group intervention, provided by a relevant practitioner.

Guidelines with an emphasis on interventions have been created for specific academic fields, such as the guide for reporting nursing interventions developed by Conn and Groves (2011). The WIDER (Workgroup for Intervention Development and Evaluation Research) recommendations for reporting behaviour change interventions, with the focus on clinical style interventions [5], and CReDECI 2 (Criteria for Reporting the Development and Evaluation of Complex Interventions in healthcare) to guide reporting the development and evaluation of complex interventions [6]. We established that while there are a number of reporting guidelines addressing various aspects of studies and study designs, guidance to facilitate the complete description of the intervention is not provided in existing guidance, except for the template for intervention description and replication (TIDieR).

Comparison of PHP intervention studies with original TIDieR checklist

We undertook an assessment exercise with a sample of PHP intervention studies to establish what information is not captured, or not captured well, by using the original TIDieR checklist.

We identified a diverse range of PHP interventions, including studies of tobacco and alcohol regulation, health service reorganisation, welfare system changes, conditional incentives for behaviour change and environmental or infrastructural improvement programmes. Through a combination of purposively searching literature and the project team members' personal libraries, we sought examples for the different types of PHP interventions (legal, fiscal, structural, organisational, environmental, and policy interventions). Several members of the project team have extensive knowledge of PHP interventions and strong awareness of PHP diversity.

Four authors (MC, SVK, RA, PC) independently abstracted intervention descriptions from published evaluations. The TIDieR checklist was then used to assess: what the checklist captures well, what is partially captured (e.g. by minor alteration of checklist terminology or examples provided), and what details were not captured by using the original checklist. The findings were used for discussion within the team. By considering the reasons why particular interventions were clearly suited to TIDieR or TIDieR-PHP, and those where it was unclear, attributes important to include in TIDieR-PHP emerged. Intervention characteristics that did not easily fit the existing checklist, such as the policy and organisational setting of the intervention and the 'intensity of exposure' involved in interventions that offer transfer payments or impose sanctions. A draft reporting checklist was created through an iterative process of incorporating these characteristics into the checklist and testing them against additional interventions.

Modified Delphi consultation

We asked researchers with PHP expertise to pilot the draft reporting checklist and used their feedback to further refine the draft checklist prior to undertaking the Delphi survey. The online survey was a modified Delphi, with Round One presenting the participants with a draft list of items rather than an entirely open round of idea gathering[7]. This modified method of the Delphi exercise has been used previously to develop reporting guidelines[8 9]. A modified Delphi was appropriate for our project as the aim was to adapt the original TIDieR checklist. The online survey platform was developed by the MRC/CSO Social and Public Health Sciences Unit, University of Glasgow web developer, and the survey was approved by the University of Glasgow College of Social Sciences Ethics Committee.

Delphi participants

The Delphi process intended to seek the views of a wider range of experts. All invited Delphi participants were funders, editors, or researchers with public health expertise. Prior to including

individuals on the list of Delphi invitees, we checked that they were funders, editors, reviewers, or researchers specifically of PHP interventions, through authorship of relevant peer-reviewed articles or involvement in reviewing or funding PHP interventions. We were intent on Delphi participants with knowledge of the implications of reporting PHP interventions rather than one-to-one, or small group, clinical-style delivered interventions. We invited 108 individuals, including participants from low and middle-income countries, to facilitate international applicability of the reporting guideline.

Knowledge within the TIDieR-PHP project team was used to compile an initial list of people with professional expertise of social and public health interventions. We also invited participants via presentations at two research conferences (EQUATOR-Reward, Edinburgh, October 2015, and the Lancet Public Health Sciences conference, London, November 2015). Potential participants within the research and social and public health community were contacted via their publically available work email addresses and invited to participate in the online survey. The email outlined the purpose of the survey and provide a link to the online survey, created by the Social and Public Health Sciences Unit web developer. The introductory page of the survey provided a clear plain language statement explaining the survey and a pdf of full participant information details relating to the survey. Other than job category, no personal information was collected. Participants were asked to tick a box on the introductory page of the survey to state they consented to participating in the study. The box required to be checked to enable the survey to commence. The participant information sheet included explanation that: the information provided would be used anonymously; the survey conducted using a secure server; the results saved onto an encrypted computer and stored according to MRC guidelines for ten years following completion of the study; and that every effort would be made to maintain confidentiality.

Round One

The first round of the survey listed 12 draft items. Each item was an adaptation of the original TIDieR item, revised to fit PHP interventions, based on the comparison exercise described above. We invited the participants to rate the items on a four-point Likert Scale (essential, desirable, possible, omit), and comment on the suitability of each item and its wording. The rating scale was successfully used in the development of TIDieR, and has been used to develop other reporting guidelines (e.g. PRISMA for Abstracts)[9]. The survey sought suggestions on the content and grouping of draft checklist items and sub points of each item, specifically whether the new checklist items sufficiently captured the pertinent details of population health and policy interventions. The survey was available for four

weeks. A reminder email was sent to all respondents, unless they had indicated that they did not wish to be involved in the consultation, two weeks after the initial invitation.

The introductory page of the survey provided a clear plain language statement explaining the survey and a link to a pdf of further information about the survey. Other than job category (Tables 1 and 2 provides participants' self-reported role), no personal information was collected. To open the survey, participants were required to tick a box on the introductory page to state that they consented to participating in the study.

After Round One of the survey closed, the de-identified responses were exported into Excel and the quantitative data (rating of each item) and the qualitative data (free text comments for each item) were collated. Round One had a 47% response rate (51/108). The project team met to discuss the results and adapt the checklist items in accordance with the ratings of the items and the qualitative data provided. The qualitative feedback on the items, their grouping, and wording, was as important as the rating of each item, therefore, we did not set a specific agreement level a priori. All of the items that were presented in Part A of Round Two (items to be included) had an 'essential' rating of 70% or more, except item 5 'who provided'. For this item the rating was split between 'essential' (55%) and 'desirable' (25%), with 12% 'possible', 0% 'omit', and 8% missing (Table 3). The open-ended responses also favoured inclusion.

Table 1 Delphi respondents' self-reported role description

**Background**	**n**
Funder	2
Publisher/editor	3
Knowledge broker	1
Policymaker	1
Intervention researcher	16
Qualitative researcher	4
Quantitative researcher	7
Mixed methods researcher	7
Statistician	1
Systematic reviewer	6
Missing	3
Total	51

Table 2 Delphi respondents' institution description

**Background**	**n**
University	41
Non-profit organisation	4
For profit organisation	2
Non-University research institute	1

Missing	3
total	51

Table 3 Summary results for Round One of Delphi survey

Item	Item label	Essential (n)	Desirable (n)	Possible (n)	Omit (n)	Missing (n)	Essential (%)	Essential + Desirable (%)
1	Brief name	37	7	5	1	1	73%	86%
2	Why	40	7	2	1	1	78%	92%
3	What - materials	36	11	1	0	3	71%	92%
4	What and how	36	10	2	0	3	71%	90%
5	Who provided	28	13	6	0	4	55%	80%
6	(how)	30 Incorporate into 'What and How'		14 separate 'how' item		7		
7	Where	36	9	3	0	3	71%	88%
8	When	41	3	4	0	4	80%	86%
9.1	Planned variation	35	9	2	1	4	69%	86%
9.2	Unplanned variation	31	10	5	1	4	61%	80%
10	Modifications	30	10	3	3	5	59%	78%
11.1	How well	23	13	10	0	5	45%	71%
11.2	Implemented as intended	28	9	6	2	6	55%	73%

Round Two

A revised version of the TIDieR-PHP checklist was developed into Round Two of the online survey. All Round One Delphi participants were invited to participate in Round Two, unless they had requested to withdraw from the project. Round Two comprised three parts. Part A contained the proposed items to include in the checklist unless strong objection was received in this round. The participants did not need to rate those items again, but were invited to provide comments. Part B contained items that the result of Round One indicated required substantial revision of intention, explanation or wording. The rating results from Round One were reported and a summary of the issues raised was provided for each of the revised items. Participants were invited to rate these items on the four-point scale as in Round One and again, to provide comments. Part C consisted of items that, based on Round One results, were likely to be omitted from the checklist, as the content had been incorporated into the remaining items. The rating results from Round One were reported and a summary of the issues raised was provided for each items. Participants were asked to comment whether they objected to these omissions. Round Two had a 71% response rate (36/51).

Finalising the TIDieR-PHP checklist

The results of Round Two found high agreement for the suggested checklist items (Table 4). The levels of agreement in combination with the rich qualitative data provided in Rounds One and Two on the content and wording of the items, led the team to conclude that a further round of the Delphi exercise was not required. The anonymised qualitative data from both Rounds were collated and circulated to the TIDieR-PHP team. The qualitative data from the Delphi, in combination with the project team members' experience of conducting, evaluating, reviewing PHP interventions, and development of the original TIDieR guidance (TH), was used to bring collate the TIDieR-PHP guidance. The TIDieR-PHP team convened via remote meetings, and through discussion and iteration finalised the wording of the checklist items and explanations.

Table 4 Summary of results for Round Two of Delphi survey

**Part A**<br>**Items 1(brief name), 2(why), 3(what – materials), 4(what and how), 5(who provided), 7(where), 8(when)**<br>**To be included in guidelines**		Support for the inclusion of these items.<br>Comments suggest some clarification of item wording will be useful.			
**Part B**<br>**Item**		**Yes (agree) (n)**	**No (disagree) (n)**	**Missing (n)**	**Yes (agree) (%)**
9.1	Planned variation	28	7	1	78%
9.2	Unplanned variation	29	4	3	81%
11.1	How well	29	6	1	81%
11.2	How well – delivery	33	2	1	92%
**Part C**<br>**Items 6 (how), 10(modifications)**<br>**To be merged with other items**		Support for merging these item into the appropriate item.<br>Comments suggest this should include clear signposting of the merged items.			

Intervention categories

During the development of the TIDieR-PHP checklist, it became apparent that providing examples of different types of PHP interventions may help checklist users to identify what information may be useful to include. Therefore, TIDieR-PHP includes additional signposting in relation to some common characteristics of population health and policy interventions. These categories and accompanying examples are listed in Table 5.

It should be noted that these examples are intended as suggestions and this list should not be considered definitive or exhaustive. The intention is to offer general examples to encourage TIDieR-PHP users to consider the types of interventions that would benefit from using the TIDieR-PHP checklist. It is possible that PHP interventions may have features covered by more than one of these categories.

Table 5 TIDieR-PHP Examples of intervention categories

Categories	Examples
Incentive-based interventions	incentives for smoking cessation in pregnancy
Regulatory interventions	air pollution controls; regulation of the use of trans fats in processed foods
Infrastructural interventions	provision of clean drinking water or improved sanitation; neighbourhood regeneration
Material benefit interventions	free school meals; welfare benefits
Societal or major policy reforms	mass privatisation in post-communist countries, integration of health and social care services
Taxation and pricing interventions	taxes on specific foods or food constituents; minimum unit pricing for alcohol
Social marketing, communications, mass media interventions	information adverts on benefits of physical activity
Settings-based, choice architecture, nudge interventions	displaying healthier food options more prominently

Strengths and limitations

While the guideline-development recommendations [1] suggest that a comprehensive literature review can be helpful in the process at this stage, as we had established that there was currently no reporting guideline, the most appropriate method to identify what was not being captured was to conduct an assessment exercise using PHP interventions and the TIDieR checklist. A possible limitation is the relatively low response rate to the Delphi survey. However, we believe the number of participants is less important than the expertise of those who take part. We are confident that the TIDieR-PHP participants had the range of expertise we required. Strengths of this project are that this is the first guideline to provide guidance for reporting PHP interventions. The project team included the lead author of the original TIDieR guideline. The guidance was developed by the use of a two-round Delphi exercise to gather consensus on the PHP intervention reporting guideline, and all the Delphi participants were public health funders, editors, or public health researchers of PHP interventions. Future research in which the TIDieR-PHP guideline is formally evaluated by authors of PHP evaluative studies as they are writing protocols or papers would be useful.

References

1. Moher D, Schulz KF, Simera I, Altman, DG. Guidance for developers of health research reporting guidelines. *PLoS Med* 2010;7(2):e1000217
2. Schulz KF, Altman DG, Moher D, *CONSORT Group*. CONSORT 2010 statement: updated guidelines for reporting parallel group randomised trials. *BMJ* 2010;340:c332 doi: 10.1136/bmj.c332.
3. Chan A-W, Tetzlaff JM, Altman DG, Laupacis A, Gøtzsche PC, Krleža-Jerić K, et al. SPIRIT 2013 statement: defining standard protocol items for clinical trials. *Ann Intern Med* 2013;158(3):200-07 doi: 10.7326/0003-4819-158-3-201302050-00583.
4. Des Jarlais DC, Lyles C, Crepaz N. Improving the reporting quality of nonrandomized evaluations of behavioral and public health interventions: the TREND statement. *Am J Public Health* 2004;94(3):361-66.
5. Albrecht L, Archibald M, Arseneau D, Scott SD. Development of a checklist to assess the quality of reporting of knowledge translation interventions using the Workgroup for Intervention Development and Evaluation Research (WIDER) recommendations. *Implement Sci* 2013;8:52 doi: 10.1186/1748-5908-8-52.
6. Mohler R, Kopke S, Meyer G. Criteria for Reporting the Development and Evaluation of Complex Interventions in healthcare: revised guideline (CReDECI 2). *Trials* 2015;16:204 doi: 10.1186/s13063-015-0709-y.
7. Murphy MK, Black NA, Lamping DL, *McKee C, Sanderson C, Askham J,* et al. Consensus development methods, and their use in clinical guideline development. Health Technol Assess 1998;2(3): 1-88.
8. Hoffmann TC, Glasziou PP, Boutron I, Milne R, Perera R, Moher D, Altman D, et al. Better reporting of interventions: template for intervention description and replication (TIDieR) checklist and guide. *BMJ* 2014;348
9. Beller EM, Glasziou PP, Altman DG, Hopewell S, Bastian H, Chalmers I, et al. PRISMA for Abstracts: Reporting Systematic Reviews in Journal and Conference Abstracts. *PLoS Med* 2013;10(4):e1001419 doi: 10.1371/journal.pmed.1001419.
