# Supplementary material for: TIDieR-PHP: a reporting guideline for population health and policy interventions
Source: BMJ. 2018 May 16;361:k1079. doi: 10.1136/bmj.k1079 (PMC5954974; doi:10.1136/bmj.k1079)
Supplement: Supplementary file 2 — Population health and policy intervention examples and citation details [file camm041840.ww2.pdf]

TIDieR-PHP: a reporting guideline for population health and policy interventions, explanation and elaboration

Supplementary file 2: Population health and policy intervention examples and citation details

Table 1: PHP intervention examples

Item	Item with examples (citations available in Table 2 below)
**1 Brief name**	**Provide the name or a phrase that describes the intervention**<br><br>**1a** The Swansea Bureau: A model of diversion from the Youth Justice System<br><br>**1b** Effectiveness of the Healthy Lifestyles Programme (HeLP) to prevent obesity in UK primary-school children: a cluster randomised controlled trial
**2 Why**	**Describe the logic, mechanisms or rationale of the intervention clearly linking intervention elements to the expected effects on immediate or longer term outcomes (or both)**<br><br>**2a** "The guiding rationale for UCTs [unconditional cash transfers] is that poor people are rational economic actors but merely lack the resources (money) to realize preferred investment levels. In other words, they possess the knowledge to make the wisest spending decisions that would improve their livelihoods, but they cannot do so because of financial constraints [reference provided]. By providing additional income in the form of cash transfers, households' credit constraints are freed up, allowing them invest in things like education. … Separate from theoretical arguments, an important reason for UCTs in SSA [sub-Saharan Africa] is that there are significant barriers to attaching conditions for both targeted populations and implementers." (page 64)<br><br>**2b** "We have attempted to capture how we think HeLP engages children and families in the HeLP Process Model (Figure 2). This model attempts to represent the mediating variables related to HeLP within the IMB framework, indicating the feedback loops which appear to strengthen relationships and engagement with HeLP over time in order to create behaviour change." (page 13)<br><br>**2c** "Seguro Popular consists of health policies [reference provided] and priorities, [reference provided] including entitlements for affiliated families, well-defined benefits packages including coverage for 266 unique health interventions, 312 medicines, increased funds to state health ministries proportional to the number of Seguro Popular-affiliated families, federal funds for personal and non-personal health services, and creation of special federal funds for catastrophic medical expenditures associated with certain diseases.6 By linking federal support to medical facility quality, Seguro Popular aims to strengthen an accreditation system for health clinics and hospitals. When rollout is complete, Seguro Popular is intended to increase total health spending in Mexico by a full percentage point of gross domestic product (from 5·6% in 2002)." (page 1447)
**3 What - materials**	**Describe any materials used in the intervention (including online appendices or URLs for further details), e.g.:**

	informational materials (may include those provided to recipients of the intervention or in training of intervention providers) **nature and value of any benefit provided (e.g. cash, voucher, meal)** **any physical resources or infrastructure provided as part of the intervention** <br>**3a** “the food demonstration was accompanied by distribution of traditional recipe cards plus health promoting cards, the size of a business card and billed as such with the *tienda*’s name. … The two *tiendas* also received posters, price tags, shelf tags, streamers (aka *papel picado;* a traditional Mexican form of art that consists of a long string of paper-cut designs, commonly used at celebrations such as birthdays), nutrient information tables for produce, and other point-of-purchase materials showcasing the fruit or vegetable promoted that week.” (page 5)<br><br>**3b** “Voucher incentive payments. Those allocated to the intervention group will be offered financial incentives - Love2Shop vouchers - that can be redeemed in a wide array of UK shops but not used for the purchase of cigarettes or alcohol. The total amount available is £400 if women remain abstinent at each monitoring point” (page 5)<br><br>**3c** “According to JSY’s guidelines, after delivery in a government or accredited private health facility, eligible women would receive 600 Indian rupees (US$13·3) in urban areas and 700 rupees ($15·6) in rural areas. In ten high-focus states (Uttar Pradesh, Uttaranchal, Bihar, Jharkhand, Madhya Pradesh, Chhattisgarh, Assam, Rajasthan, Orissa, and Jammu and Kashmir) with low in-facility birth coverage, all women irrespective of socioeconomic status and parity are eligible for the cash benefit. (page 2009)<br><br>**3d** “In Salvador, construction included the laying of more than 2000 km of sewer pipes, building 86 pumping stations, and connection of more than 300 000 households to the sewerage network.” (page 1623)
**4 What and How**	**Describe how the intervention was planned, established, and intended to be delivered. Depending on the type of intervention, it may be useful to consider:** **how sources of funding for the intervention and the service providers were obtained, how users were enrolled and the service delivered** **how any payments were made or benefits delivered, how qualifying conditions were implemented** **the entity being regulated, the scope of the regulation, permitted level of use; procedures for monitoring or enforcing compliance, and any sanctions for non-compliance** **how people were exposed to the intervention, whether it was provided to individuals or larger populations** **any underpinning legislation including name, date passed and legislative body** <br>**4a** “Through competitive tendering, the Department of Health identified 27 existing projects to deliver the YPDP, which received additional funding and support from the National Youth Agency, a non-governmental agency. Tenders were judged on the quality of the proposed work, local deprivation, and teenage pregnancy rates and to ensure geographical spread and ethnic/gender diversity of participants.” (page 1)

	**4b** "Salt replacement will be progressively implemented over six months in each village. The intervention will contemplate interactions with families as well as bakeries, community kitchens, food vendors including street vendors, and restaurants. Ideally, replacement will require a complete exchange of ordinary salt." (page 3)<br><br>**4c** "Women will receive incentive payments outlined below: £50 for attending a face-to-face appointment with their NSPS adviser and setting a quit date; £50 if quit 4 weeks after their quit date corroborated by a carbon monoxide breath test result less than 10 ppm collected by a research nurse; £100 if quit after 12 weeks corroborated by a carbon monoxide breath test collected by a research nurse; £200 if they self-report quit for at least 2 months when contacted for primary outcome assessment by the Helpline at 34 to 38 weeks gestation. This will be corroborated by a carbon monoxide breath test result less than 10 ppm collected by a research nurse." (page 6)<br><br>**4d** "The primary feature of the [Licensing] Act was the removal of fixed closing times that limited the temporal availability of alcohol at licensed outlets. Under the previous system, premises were allowed to serve alcohol between the hours of 11:00 and 23:00 (22:30 on a Sunday) with a standard license, and between 11:00 and 02:00 (03:00 in London) with a 'Special Hours Certificate'. The new legislation allowed greater flexibility in licensing, promoting a system of 'natural staggering', which was believed to be better suited to modern consumers and retailers" (page 2)<br><br>**4e** "The *Alcohol etc. (Scotland) Act 2010* ('Alcohol Act'), implemented on 1 October 2011, introduced new regulations concerning the sale of alcohol in the off-trade [8]. These regulations limited alcohol promotions within retail stores to a single area and introduced a ban on quantity-based price discounts. This latter aspect of the legislation was considered to be its main component, and prevented off-trade retailers from providing multi-buy promotions such as 'three for the price of two' or 'three bottles for £10'; however, straight discounting of products remained permissible. The intention was to restrict incentives encouraging consumers to purchase more alcohol than they may otherwise have bought, and thereby reduce consumption." (page 2036)
**5 Who provided**	**Describe the provider of the intervention, including legal status and powers, field organisations and staff responsible for planning, implementation, monitoring and enforcement. Where relevant, describe intervention provider expertise and training (general or specific to the intervention)**<br><br>**5a** "The Bureau is intended as a new approach to diversion, utilising inter-agency partnership (in line, in fact, with YJB guidance) between South Wales Police and Swansea Youth Offending Service (YOS), supported by the wider Safer Swansea Partnership. It operates within a formal partnership agreement, which specifies the roles of the respective local agencies, sets out agreed referral eligibility criteria and clarifies decision-making responsibilities." (page 171)<br><br>**5b** "The Well London project is a four-year programme that uses a community development approach to deliver a set of complex health interventions aimed at improving HE, HPA and MH in the most deprived neighbourhoods of 20 London Boroughs. The project is led by The Greater London Authority with the London Health Commission and a consortium of partners including Groundwork London, the London

	Sustainability Exchange (LSx), the Central YMCA, the University of East London, the South London and Maudsley NHS Foundation Trust (SLaM) and the Arts Council for England. These partners together form the Well London Alliance. The project was launched at the end of 2007 and the interventions, developed in detailed consultation with communities, local authorities (LAs), Primary Care Trusts (PCTs) and London strategic bodies, will be delivered in two phases (covering 9 and 11 Boroughs respectively) with a 6 months gap between them." (page 2)<br><br>**5c** "In alignment with Queensland Health Multicultural Guidelines, all of the MHW's [multicultural health workers] were of Maori or Pacific Islander descent and lived within the communities they serviced, including representatives from seven communities: Cook Islands, Fiji, Fiji Indian, Maori, Samoan, Tongan and Papua New Guinean. … The program activities were implemented in schools with high numbers of children from Maori and Pacific Islander backgrounds (ranging from 10-90% of the total school population)" (page 3)<br><br>**5d** "Five day residential youth-friendly clinic staff training for at least one nurse in each clinic with refresher training after 2 years. On-site training was provided for remaining clinic staff. Monthly support visits by project staff when key features of clinic accessibility were assessed and on-site training tailored to any deficiencies noted. Standards for youth-friendly services provision were developed by clinic nurses, who are independently assessed against these standards at 6 monthly intervals. Prizes are awarded to clinics performing well. Each clinic receives detailed independent feedback on their performance at these standard assessments. The district nursing leadership is an integral part of this process." (page 1238)
**6 Where**	**Describe the type of location (e.g. school, community centre) and the geographical scope of the intervention (e.g. national, regional, city-wide). Where relevant, describe the historical, cultural, socioeconomic, or political background to the intervention**<br><br>**6a** "Acayucan is one of Mexico's 56 metropolitan areas encompassing three municipalities with a combined population of 105,000 (INEGI, 2007). The city has a central core where most streets have been paved, and outer sections where street pavement is gradually rolled out. Residences are built and inhabited long before streets are paved. … The intervention consists of first-time asphalting of residential nonarterial streets" (page 255)<br><br>**6b** "This study involves inhabitants from Tumbes, a department located in the north coastal region of Peru on the border with Ecuador. The semi-urban area of Tumbes consists of more than 100 villages of varying size with an approximate total population of 80,000. It is comprised of a large 'mestizo' – mixed of European and Amerindian ancestry – population, and the traditional agricultural and fishing landscape has become intermixed with rapidly growing urban sections. Illiteracy rate is around 10%, and 50% of the inhabitants have no health insurance. … [T]here are both challenges and opportunities related to salt substitution in Peru. A previous successful public health measure involved promoting iodine-supplemented salt as a vehicle to prevent iodine deficit disorders, such as goiter, and in commercial spheres, the slogan "*Consuma salud… consuma sal*", or "Buy health… buy salt", is being promoted. This is a challenge for our intervention because salt has been marketed as a positive health

	measure, and we now propose to promote the replacement of regular salt with a substitute." (page 6)
**7 When and How often**	**Describe when the intervention was implemented, how long it remained in place, and if applicable, the number, duration and scheduling of occasions**<br><br>**7a** "The community intervention comprises two modules (eleven 3-h [hour] sessions for each module)" (page 1238)<br><br>**7b** "As part of the 1991 reforms larger general practices were given the option to become fundholders [reference provided]. Fundholding practices were given a budget by their local HA [Health Authority] to purchase certain types of elective secondary care procedures (chargeable electives) from hospitals. … No new fundholders were allowed from the start of the financial year April 1998 and fundholding was abolished in April 1999. After April 1999 no practice had budgetary responsibility for the costs of secondary care for its patients." (page 450)
**8.1 Planned variation**	**Describe and provide the reason for any variation or tailoring that was <u>planned</u> or allowed for in the design of the intervention. Examples include differences between locations, geographical areas, population subgroups or over time**<br><br>**8.1a** "The key elements of the whole school component were the establishment and support of a school based adolescent health team; the identification of risk and protective factors in each school's social and learning environment from student surveys; and, using these data, the identification of effective strategies to address these issues." (page 998)<br><br>**8.1b** "The researchers asked permission from community leaders and engaged them in discussion of baseline evidence. Facilitators convened and ran intervention design groups—8-10 people, usually separately for men and women—to discuss survey results, cost implications, and specific prevention strategies in each community. The exact process for convening the groups varied from place to place, with some participants suggested by community leaders, some being key figures like school teachers, and some identified by door to door invitation … Communities opted for a range of activities to share basic information on the mosquito life cycle and how to interrupt it (emptying, brushing/scrubbing the interior walls of, or covering receptacles hosting mosquito eggs or larvae); community events to raise awareness, like puppet shows and basketball tournaments; clean up campaigns focused on unoccupied and public premises; introduction of fish into water storage containers (Mexico only); and other activities listed in appendix 2. … Brigadistas also added interventions as their community work advanced (see appendix 2). In Nicaragua, brigadistas received no remuneration; in Mexico, they received allowances for travel, lunch, and child care on the days they worked." (page 3)<br><br>**8.1c** "Because of their local autonomy, SSLPs [Sure Start Local Programmes] do not have a "protocol" to promote adherence to a prescribed model, as do other early interventions that are known to be effective. All SSLPs are expected, nevertheless, to provide core services of outreach or home visiting; family support; support for good quality play, learning, and childcare experiences; primary and community health care; advice about child and family health and development; and support for people with special needs, including help in accessing specialised services. Community

	participation is central to the mission of these programmes, through local partnerships that bring together all people who are concerned with children in the local community, including health, social, and education services; the private sector; the voluntary sector; and parents" (page 1)
**8.2 Unplanned variation**	**Describe and provide the reason for any <u>unplanned</u> variation or modifications in the intervention (e.g. between different locations, geographical areas, population subgroups or over time) that were made after the intervention commenced**<br><br>**8.2a** "In one geographically bounded sub-community the builder carrying out the retrofit claimed to have carried out the intervention according to contractual specifications, but failed to do so, until the discrepancies were picked up by the external auditor. This contractor's deception was not detected by the householders. Consequently, in part of one community, subjects incorrectly believed they had received the intervention at the time of reporting results for follow-up." (page 2607)<br><br>**8.2b** "SSLPs [Sure Start Local Programmes] have evolved, changing their model of service delivery by becoming Sure Start Children's Centres during 2004-06.The changes included clearer specification of services, with a strong emphasis on child wellbeing and the need to reach the most vulnerable, and adjustment of service provision to the degree of family disadvantage." (page 1642)<br><br>**8.2c** "[A]n unexpected result from the interviews with parents was to add a second, parallel intervention for parents. … The original implementation plan was revised to add a modified version of the Families Matter! (FM!) program, adapted for parents of older youth than the original FM! Intervention, which focused on parents of 9- to 12-year-olds. In addition, a joint session was added to both programs in which both parents and adolescents came together for guided practice of their newly acquired conversational skills for discussions of sensitive issues within the family." (page 284)
**9.1 How well**	**Describe any strategies used or actions taken to maintain fidelity of the intervention (i.e. to ensure that the intervention was delivered as intended)**<br><br>**9.1a** "A cornerstone of the intervention is that its facilitators are very carefully selected, trained and supported. The facilitators who implement the intervention with parents go through a similarly rigorous selection process and undergo 2 weeks of residential training on the course materials and facilitation skills. Both groups are supported by the project intervention team who provide ongoing mentoring. Although the initial recruitment and training of facilitators is time consuming (and accounts for the bulk of intervention delivery costs), it is a manageable and easily replicable activity that then forms the backbone of the intervention." (page 1242)<br><br>**9.1b** "To help promote local police department cooperation, a retired state trooper was hired as the project law enforcement liaison midway into the project. Furthermore, additional funds were secured to support project-related enforcement activities. The coordination of the liaison coupled with additional funding significantly enhanced the quantity of project-related enforcement efforts." (page 5)
**9.2 How well - delivery**	**Describe fidelity of the intervention (i.e. the <u>extent</u> to which the intervention was delivered as intended)**

	**9.2a** “Early compliance with the Scottish legislation was high. Two weeks after implementation of this legislation, the levels of secondhand smoke in bars had decreased by 86%.” (page 488)<br><br>**9.2b** “The country introduced the UHC [universal health coverage] policy, originally known as the 30 baht project, in 2001. Three different schemes are in place, including, two employment-based schemes and the tax-financed Universal Coverage Scheme. The nationwide roll-out of the Universal Coverage Scheme was completed within a year, reaching a coverage of 71%. Coverage increased to 95% in 2003, and 98% by 2011 (International Health Policy Program, 2011).” (page 46)<br><br>**9.2c** “All 32 schools completed the trial. All schools in the intervention group completed or nearly completed the whole programme and the quality of delivery in all schools was at or above the established appropriate level (appendix). 629 (93%) of the 676 children in the intervention group were categorised as compliers (ie, they received at least four of the five drama sessions and the one-to-one goal-setting discussion in phase 3). No notable differences in uptake were seen between the two cohorts (appendix). 353 (52%) of the 676 children had family attending at least one parent event and 652 (96%) children set goals with the HeLP coordinator in phase 3. 411 (63%) of these 652 children had parental support, shown by a parent signature on the goal-setting sheet or written comments about how the parent would support the child in achieving their goals.” (page 38)

Table 2 Citations for guideline examples

Example	Citation
1a	Haines K, Case S, Davies K, Charles A. The Swansea Bureau: A model of diversion from the Youth Justice System. *Int J Law Crime Justice* 2013;41:167-87
1b	Lloyd J, Creanor S, Logan S, et al. Effectiveness of the Healthy Lifestyles Programme (HeLP) to prevent obesity in UK primary-school children: a cluster randomised controlled trial. *Lancet Child Adolesc Health* 2018;2:35-45.
2a	Kilburn K, Handa S, Angeles G, Mvula P, Tsoka M. Short-term impacts of an unconditional cash transfer program on child schooling: Experimental evidence from Malawi. *Econ Educ Rev* 2017;59:63-80 (page 64)
2b	Lloyd JJ, Wyatt KM. Qualitative findings from an exploratory trial of the Healthy Lifestyles Programme (HeLP) and their implications for the process evaluation in the definitive trial. *BMC Public Health* 2014;14:578 (page 13)
2c	King G, Gakidou E, Imai K, et al. Public policy for the poor? A randomised assessment of the Mexican universal health insurance programme. *Lancet*;373:1447-54 (page 1447)
3a	Ayala GX, Baquero B, Laraia BA, Ji M, Linnan L. Efficacy of a store-based environmental change intervention compared with a delayed treatment control condition on store customers’ intake of fruits and vegetables. *Public Health Nutr* 2013;16:1953-60 (page 5)
3b	Tappin DM, Bauld L, Tannahill C, et al. The cessation in pregnancy incentives trial (CPIT): study protocol for a randomized controlled trial. *Trials* 2012;13:113 (page 5)
3c	Lim SS, Dandona L, Hoisington JA, et al. India's Janani Suraksha Yojana, a conditional cash transfer programme to increase births in health facilities: an impact evaluation. *Lancet*;375:2009-23 (page 2009)

3d	Barreto ML, Genser B, Strina A, Assis AMO, Rego RF, Teles CA, et al. Effect of city-wide sanitation programme on reduction in rate of childhood diarrhoea in northeast Brazil: assessment by two cohort studies. *Lancet* 2007;370:1622-8 (page 1623)
4a	Wiggins M, Bonell C, Sawtell M, et al. Health outcomes of youth development programme in England: prospective matched comparison study. *BMJ* 2009;339:b2534 (page 1)
4b	Bernabe-Ortiz A, Diez-Canseco F, Gilman RH, et al. Launching a salt substitute to reduce blood pressure at the population level: a cluster randomized stepped wedge trial in Peru. *Trials* 2014;15:93 (page 3)
4c	Tappin DM, Bauld L, Tannahill C, et al. The cessation in pregnancy incentives trial (CPIT): study protocol for a randomized controlled trial. *Trials* 2012;13:113 (page 6)
4d	Humphreys DK, Gasparrini A, Wiebe DJ. Evaluating the impact of Florida's "Stand Your Ground" self-defense law on homicide and suicide by firearm: an interrupted time series study. *JAMA Intern Med* 2017;177:44-50 (page 2)
4e	Robinson M, Geue C, Lewsey J, et al. Evaluating the impact of the alcohol act on off-trade alcohol sales: a natural experiment in Scotland. *Addiction* 2014;109:2035-43 (page 2036)
5a	Haines K, Case S, Davies K, et al. The Swansea Bureau: A model of diversion from the Youth Justice System. *Int J Law Crime Justice* 2013;41:167-87 (page 171)
5b	Wall M, Hayes R, Moore D, et al. Evaluation of community level interventions to address social and structural determinants of health: a cluster randomised controlled trial. *BMC Public Health* 2009;9:207 (page 2)
5c	Mihrshahi S, Vaughan L, Fa'avale N, et al. Evaluation of the Good Start Program: a healthy eating and physical activity intervention for Maori and Pacific Islander children living in Queensland, Australia. *BMC Public Health* 2017;17:77 (page 3)
5d	Cowan FM, Pascoe SJS, Langhaug LF, et al. The Regai Dzive Shiri Project: a cluster randomised controlled trial to determine the effectiveness of a multi-component community-based HIV prevention intervention for rural youth in Zimbabwe – study design and baseline results *Trop Med Int Health* 13: 1235-1244 (page 1238)
6a	Gonzalez-Navarro M, Quintana-Domeque C. Paving streets for the poor: Experimental analysis of infrastructure effects. *Rev Econ Stat* 2016;98:254-67 (page 255)
6b	Bernabe-Ortiz A, Diez-Canseco F, Gilman RH, et al. Launching a salt substitute to reduce blood pressure at the population level: a cluster randomized stepped wedge trial in Peru. *Trials* 2014;15:1 (page 6)
7a	Cowan FM, Pascoe SJS, Langhaug LF, et al. The Regai Dzive Shiri Project: a cluster randomised controlled trial to determine the effectiveness of a multi-component community-based HIV prevention intervention for rural youth in Zimbabwe – study design and baseline results *Trop Med Int Health* 13: 1235-1244 (page 1238)
7b	Dusheiko M, Gravelle H, Jacobs R, et al. The effect of financial incentives on gatekeeping doctors: evidence from a natural experiment. *J Health Econ* 2006;25(3):449-78 (page 450)
8.1a	Bond L, Patton G, Glover S, et al. The Gatehouse Project: can a multilevel school intervention affect emotional wellbeing and health risk behaviours? *J Epidemiol Community Health* 2004;58:997-1003 (page 998)
8.1b	Andersson N, Nava-Aguilera E, Arosteguí J, et al. Evidence based community mobilization for dengue prevention in Nicaragua and Mexico (Camino Verde the Green Way): cluster randomized controlled trial. *BMJ* 2015;351:h3267 (page 3)
8.1c	Belsky J, Melhuish E, Barnes J, et al. Effects of Sure Start local programmes on children and families: early findings from a quasi-experimental, cross sectional study. *BMJ* 2006; 332:1476 (page 1)

8.2a	Howden-Chapman P, Crane J, Matheson A, et al. Retrofitting houses with insulation to reduce health inequalities: aims and methods of a clustered, randomised community-based trial. *Soc Sci Med* 2005;61:2600-10 (page 1642)
8.2b	Melhuish E, Belsky J, Leyland AH, et al. Effects of fully-established Sure Start Local Programmes on 3-year-old children and their families living in England: a quasi-experimental observational study. *Lancet* 2008;372:1641-47 (page 1642)
8.2c	Lawrence JS, Seloilwe E, Magowe M, et al. Cross-Cultural Adaptation of an Adolescent HIV Prevention Program: Social Validation of Social Contexts and Behavior Among Botswana Adolescents. *AIDS Educ Prev* 2013;25:269-86 (page 284)
9.1a	Cowan FM, Pascoe SJS, Langhaug LF, et al. The Regai Dzive Shiri Project: a cluster randomised controlled trial to determine the effectiveness of a multi-component community-based HIV prevention intervention for rural youth in Zimbabwe – study design and baseline results *Trop Med Int Health* 13: 1235-1244 (page 1242)
9.1b	Flewelling RL, Grube JW, Paschall M, et al. Reducing Youth Access to Alcohol: Findings from a Community-Based Randomized Trial. *Am J Community Psychol* 2013;51:264-77 (page 5)
9.2a	Pell JP, Haw S, Cobbe S, et al. Smoke-free legislation and hospitalizations for acute coronary syndrome. *N Engl J Med* 2008;359:482-91 (page 488)
9.2b	Rieger M, Wagner N, Bedi AS. Universal health coverage at the macro level: Synthetic control evidence from Thailand. *Soc Sci Med* 2017;172:46-55 (page 46)
9.2c	Lloyd J, Creanor S, Logan S, Green C, Dean SG, Hillsdon M, et al. Effectiveness of the Healthy Lifestyles Programme (HeLP) to prevent obesity in UK primary-school children: a cluster randomised controlled trial. *Lancet Child Adolesc Health* 2018;2:35-45. (page 38)
